# Mental Fatigue Might Be Not So Bad for Exercise Performance After All: A Systematic Review and Bias-Sensitive Meta-Analysis

**DOI:** 10.5334/joc.126

**Published:** 2020-10-09

**Authors:** Darías Holgado, Daniel Sanabria, José C. Perales, Miguel A. Vadillo

**Affiliations:** 1 Centro de Investigación, Mente, Cerebro y Comportamiento (CIMCYC), Universidad de Granada, ES; 2 Departamento de Psicología Experimental, Universidad de Granada, ES; 3 Departamento de Psicología Básica, Universidad Autónoma de Madrid, ES

**Keywords:** Cognitive Control, Statistical analysis, Executive functions, mental effort

## Abstract

There is an ongoing debate in the scientific community regarding whether a state of mental fatigue may have a negative effect upon a range of objective and subjective measures of human performance. This issue has attracted attention from several fields, including sport and exercise sciences. In fact, a considerable body of literature in the sport science field has suggested that performing a long and demanding cognitive task might lead to a state of mental fatigue, impairing subsequent exercise performance, although research in this field has shown contradictory results. Here, we performed a meta-analysis to investigate these inconsistent findings. The analysis yielded small-to-medium effects of mental fatigue on exercise performance, *d*_z_ = 0.50, and RPE, *d*_z_ = 0.21. However, a three-parameter selection model also revealed evidence of publication or reporting biases, suggesting that the bias-corrected estimates might be substantially lower (0.08 and 0.10, respectively) and non-significant. In sum, current evidence does not provide conclusive support for the claim that mental fatigue has a negative influence on exercise performance.

## Introduction

To achieve their goals, athletes must temper the urge to stop or reduce exercise intensity, despite the growing feeling of fatigue, effort or pain (Audiffren & André, 2015; Verhoeven et al., 2011). Exercise indeed requires controlling and monitoring afferent feedback from the muscular and cardiopulmonary systems to the brain, which can directly or indirectly affect effort regulation (Roelands, De Koning, Foster, Hettinga, & Meeusen, 2013). This is in line with the view of exercise as a complex goal-directed behavior that involves bottom-up and top-down processing (Baron, Moullan, Deruelle, & Noakes, 2011; Holgado & Sanabria, 2020; Proske & Gandevia, 2012), and highlights the relevance of cognition (specially executive function) and its neural basis to explain exercise performance, at the objective (e.g., time or distance achieved) and subjective (e.g., perceived exertion) levels.

If, as argued above, exercise performance depends on cognition, transient changes in the cognitive capacities/abilities of the athlete will probably influence their performance. Consistent with this, current research shows that performing a long and demanding cognitive task (e.g., an executive processing task) can hinder performance in subsequent physical exercise. Although there is no consensus on the ideal length of the cognitive task, most experiments exploring performance in endurance and whole-body exercises employ cognitive tasks lasting 30 minutes or longer (Pageaux & Lepers, 2018; Van Cutsem, Marcora, et al., 2017). Some studies in the ego-depletion tradition have employed somewhat similar methods with manipulations lasting less than 30 minutes, but in general they focused on physical exercises limited to a single joint or non-endurance exercises (Brown et al., 2019; Giboin & Wolff, 2019). In any case, the proponents of the psychobiological model of endurance performance (Pageaux & Lepers, 2018; Van Cutsem et al., 2017) claim that the state of mental or cognitive fatigue^1^ induced by prolonged cognitive tasks could increase the perception of effort (RPE) and therefore reduce exercise capacity, since RPE has been deemed a key variable for stopping exercise (Pageaux, 2014; Staiano, Bosio, de Morree, Rampinini, & Marcora, 2018). From this point of view, tasks with short durations might not be sufficiently demanding to have a noticeable influence on RPE and physical performance. Note that, although a consensus definition of mental fatigue has not yet been reached, in this context it is often defined as the mental state that emerges after long and demanding cognitive tasks, with a negative impact on a wide range of subjective and objective measures of human performance, including those related to physical exercise (Ishii, Tanaka, & Watanabe, 2014; Van Cutsem et al., 2017).

An illustrative example of the negative effects of mental fatigue on exercise is the study published by Marcora et al. (2009). In this study, participants in the mental fatigue condition reached exhaustion sooner in a cycling test and rated a higher RPE. Subsequently, this literature has grown considerably over the last few years, with some studies replicating Marcora et al.’s (2009) findings (e.g., Head et al., 2016; MacMahon, Schücker, Hagemann, & Strauss, 2014; Pires et al., 2018) and others reporting null effects (Holgado, Troya, Perales, Vadillo, & Sanabria, 2020; Silva-Cavalcante et al., 2018; Van Cutsem, De Pauw, et al., 2017). This discrepancy could be accounted for by several factors that deserve closer examination. First, a recent review (Pageaux & Lepers, 2018) suggests that almost 50 percent of the studies report no reliable effects of mental fatigue on different measures of exercise performance. Second, most studies on this topic are based on small samples (mean *N* = 12), which renders them underpowered to detect small but non-trivial effect sizes (Button et al., 2013). Third, although this literature has already been explored in several meta-analyses (Brown et al., 2019; Giboin & Wolff, 2019; McMorris, Barwood, Hale, Dicks, & Corbett, 2018), all of them relied on obsolete methods to detect and correct for publication bias. Analysis of publication bias seems crucial to avoid drawing premature conclusions about the presence of a true effect, since in the presence of publication bias effect sizes can be grossly overestimated, even if the null hypothesis is true.

In the present study, we tried to overcome these limitations by collating all the existing evidence in a high-powered meta-analysis, paying particular attention to the potential impact of publication and reporting biases. In addition, the current meta-analysis also explored several factors that might moderate the effects of mental fatigue on exercise, such as the cognitive demands of exercise, participants’ fitness level, or the type and duration of the cognitive task. As mental fatigue might have different impacts on (objective) physical performance and RPE, we also analysed these outcomes separately.

## Methods

### Pre-registration

The methods and planned analyses of this systematic review and meta-analysis were preregistered at PROSPERO (ref. CRD42019123250). All departures from the pre-registered protocol and their corresponding justifications are disclosed explicitly in the following sections.

### Literature Search

We used the PRISMA guidelines (Moher, Liberati, Tetzlaff, & Altman, 2009) as the basis for the procedures described herein. We carried out a literature search in April, 2019 (updated in April 2020 in order to include new studies during the review process), in Medline, Scopus and Web of Science using the following terms and Boolean operators: (“mental fatigue” or “cognitive fatigue” or “mental exertion” or “ego-depletion”) AND (“physical performance” or “exercise” or “muscle fatigue” or “sport” “or “RPE”). Searches were limited to papers published in English until April 2020. The reference lists of the retrieved studies were also reviewed to find additional studies that might not have appeared in the databases with our search terms. Additionally, we searched on Proquest and Google Scholar to identify unpublished studies meeting the inclusion criteria.

### Inclusion and Exclusion Criteria

We considered for review any study (published or not in a peer-reviewed journal) meeting the following inclusion criteria: 1) available in English; 2) randomized controlled trials; 3) participants completed a cognitive task of 30 minutes or longer prior to an exercise; 4) the main outcome was a measure of performance or perceived exertion during exercise (RPE). Among the different exercise performance measures used in this literature, we considered time to exhaustion in a physical test (in seconds or minutes, or distance completed), time-trial performance (in seconds or minutes, average power output, or average speed) or total work done. Studies were excluded following these criteria: 1) participants were symptomatic or in poor health condition, 2) the cognitive task was shorter than 30 minutes.

### Study Selection

**Figure 1:**
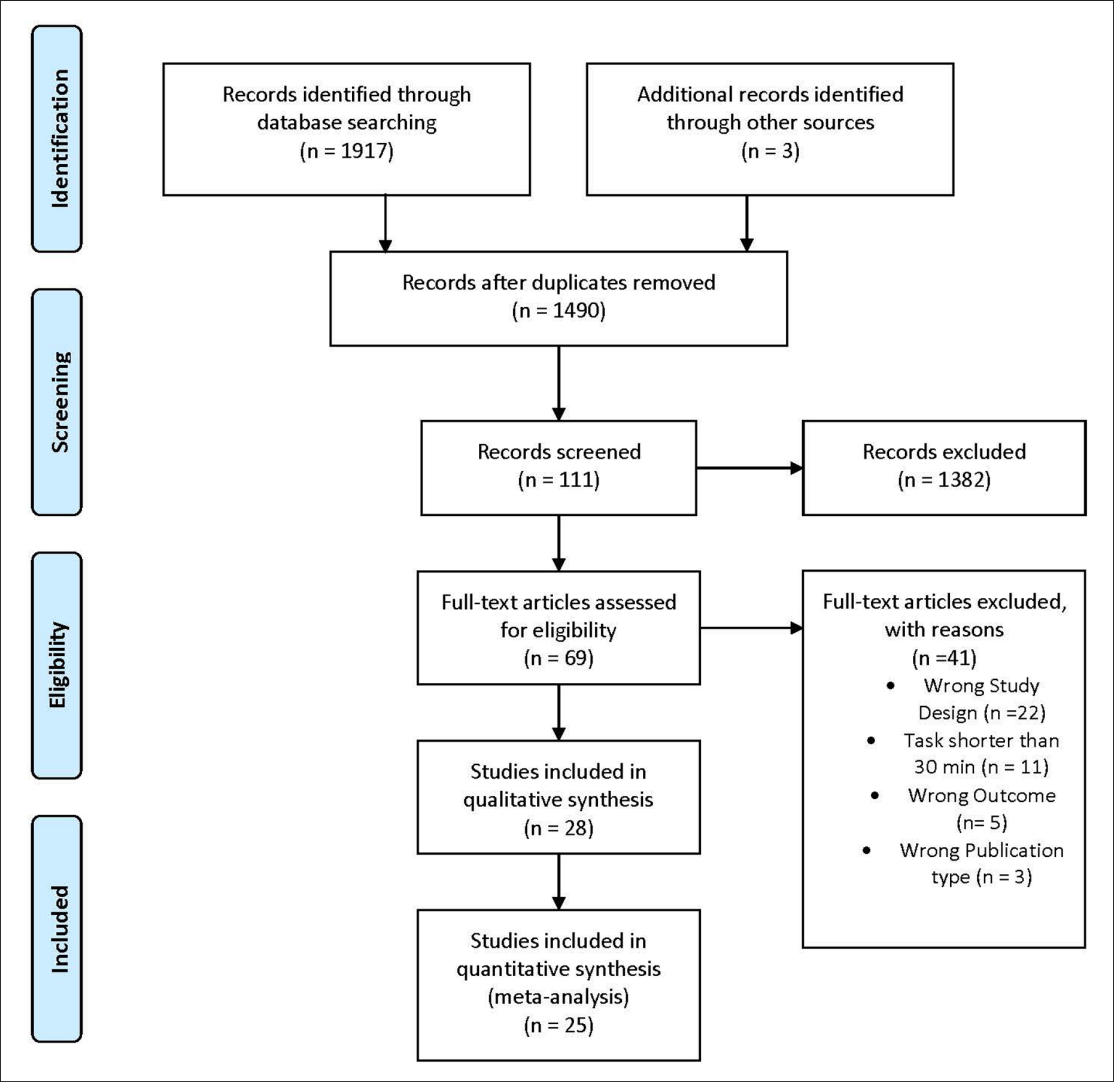
PRISMA summary of the study selection process.

Figure 1 summarizes the study selection process. The initial search returned 1,917 publications. An additional record was identified as potentially relevant after inspection of the reference list of reviews and empirical articles identified in the initial search and two more entries were included after updating the literature search. All entries were subsequently introduced in the Rayyan web service (Ouzzani, Hammady, Fedorowicz, & Elmagarmid, 2016) to facilitate the following steps of study selection. After identifying 427 duplicate articles, 111 were selected for further inspection on the basis of their title and/or abstract. When the adequacy of a study was not evident, the article was discussed by all authors to reach an agreement. The final selection of all shortlisted articles was approved by all authors. Sixty-nine full articles were assessed for eligibility and 30 of them were included in the qualitative analysis (Marcora et al., 2009; Brownsberger, Edwards, Crowther, & Cottrell, 2013; Pageaux, Marcora, & Lepers, 2013; MacMahon et al., 2014; Pageaux, Lepers, Dietz, & Marcora, 2014; Duncan, Fowler, George, Joyce, & Hankey, 2015; Martin, Thompson, Keegan, Ball, & Rattray, 2015; Azevedo et al., 2016; Badin, Smith, Conte, & Coutts, 2016; Head et al., 2016; Martin et al., 2016; Smith, Marcora, & Coutts, 2015; Smith et al., 2016; Otani et al., 2017; Van Cutsem, De Pauw, et al., 2017; Veness, Patterson, Jeffries, & Waldron, 2017; Vrijkotte et al., 2018; Brown & Bray, 2019; Clark et al., 2019; Filipas, Mottola, Tagliabue, & La Torre, 2018; Penna et al., 2018a; Penna et al., 2018b; Pires et al., 2018; Silva-Cavalcante et al., 2018; Slimani, Znazen, Bragazzi, Zguira, & Tod, 2018; Staiano, Bosio, Piazza, Romagnoli, & Invernizzi, 2018; Salam, Marcora, & Hopker, 2018; MacMahon, Hawkins, & Schücker, 2019; Holgado et al., 2020; Lopes et al., 2020). 25 of them reported sufficient information to compute at least one effect size for performance. In total, these articles contained information to compute 27 independent effect sizes for performance variables and 23 for RPE.

### Quality Assessment of Results

We used the Physiotherapy Evidence Database (PEDro) to assess the methodological quality of the studies included in the meta-analysis (Verhagen et al., 1998).

### Data Extraction

Data were extracted by DH and entered into a custom excel spreadsheet, summarized in Table 1 and publicly available at https://osf.io/s5tz6/. Given that some studies assessed two mental fatigue conditions (e.g., mental fatigue plus caffeine or mental fatigue plus heat stress; Azevedo, Silva-Cavalcante, Gualano, Lima-Silva, & Bertuzzi, 2016; Otani, Kaya, Tamaki, & Watson, 2017), we limited the extraction to the data for mental fatigue condition without any additional manipulation, to facilitate direct comparability among studies. For each study, we coded: 1) descriptive data; 2) study design; 3) type of experimental and control condition; 4) exercise protocol and type of test, and 5) main findings (mean and SD of the exercise performance and RPE outcomes; mean and SD post scores value for mental fatigue and motivation scales). Given the variety of experimental designs used in this literature, we decided to test the moderating role of a series of methodological features of these studies to explore their potential impact on the effect of mental fatigue on exercise performance. These moderators were 1) the type of outcome (performance vs. RPE); 2) exercise mode: (self-paced vs. externally-paced^2^ vs. maximal effort); 3) type of control condition (same cognitive task, but less demanding vs. neutral activity); 4) duration of the cognitive task (30–60 min vs. ≥60 min); 5) level of fitness of participants (recreational/physically active vs. well-trained/competitive vs. elite athletes); 6) whether the intervention induced the state of mental fatigue successfully (significant difference between condition: yes vs. no vs. non-reported); and 7) if the intervention reduced motivation: (significant difference between condition: yes vs. no vs. non-reported).

### Statistical Analysis

Our effect size estimate in all the quantitative analyses was the standardized mean difference, with negative sign for all the studies yielding worse performance (or higher RPE) in the experimental condition than in the control condition. In the pre-registered protocol, we specified that, for studies using within-participants designs, we would use the standard deviation of the control condition to standardize mean differences and that we would compute the variances of these effect sizes following the equations provided by Morris and DeShon (2002). This strategy was intended to improve the comparability of effect sizes in within-participants and between-groups studies. In the end, however, all the effect sizes included in the meta-analyses came from within-participants designs. Because of this, we decided to use Cohen’s *d*_z_ as our effect size estimate instead. The advantage of doing so is that *d*_z_ scores are computed on the basis of the same information that is used to test for statistical significance in these studies (i.e., a paired-samples *t*-test) and, consequently, the confidence intervals of the effect size are more consistent with the *p*-values reported in the original papers. Additionally, unlike the effect size estimate specified in the protocol, the computation of *d*_z_ does not require any knowledge of the correlation between dependent measures, which simplifies substantially the extraction of statistical information from the original studies. This decision also simplifies the estimation of the average statistical power across studies. All the analyses were performed using the *metafor* R package (Viechtbauer, 2010) and relied on random-effects models, fitted with a restricted maximum likelihood estimation algorithm. Heterogeneity across studies was quantified by means of Cochran’s Q and *I*^2^. Publication bias was tested with Egger’s test for funnel plot asymmetry and a 3-parameter selection model using the weightr package for R (Vevea & Hedges, 1995). All the data and scripts used in the following analyses are publicly available at https://osf.io/s5tz6/.

## Results

### Study characteristics

In our pre-registration form, we stated that we would include all types of exercise. However, all but four studies (two testing soccer-specific and two strength-based exercises) using a cognitive task longer than 30-min focused on endurance performance. For the sake of robustness and transparency, we ran the analysis with and without those four studies.

The main analysis on endurance performance included data from 21 studies (providing 23 statistically independent effect sizes for performance measures and 19 for RPE) and 317 participants (16% female participants). The number of participants per study ranged from 8 to 31 (mean = 13.7, SD = 6.77). In relation to the type of participants, 11 studies employed recreational sportspeople, 8 well-trained ones, and 4 elite athletes. Among the studies included in the quantitative analysis, 13 used a self-paced exercise test, 8 studies used an externally-paced exercise test and 2 anaerobic maximal effort or all-out exercise (e.g., Wingate test). Regarding the type of control condition, 21 studies used a neutral activity in the control condition (e.g., watching a documentary), whereas 2 used a less demanding cognitive task (e.g., a Stroop task without the inhibitory component). With regard to the duration of the task, in 15 studies it lasted between 30–60 minutes and in 8 studies more than 60 minutes. Fifteen studies successfully induced a state of subjective mental fatigue, whereas five did not, and three did not report it. Finally, in sixteen studies motivation was not reduced, whereas one did, and six did not report it. None of the studies were excluded based upon their PEDro score and, in general, their quality was considered good. On average, the PEDro score was M = 6.9 ± 0.76, ranging from 6 to 9. The analysis including the four remaining studies not testing endurance performance added 4 effect sizes and 64 participants.

### Overall Meta-Analysis

**Figure 2:**
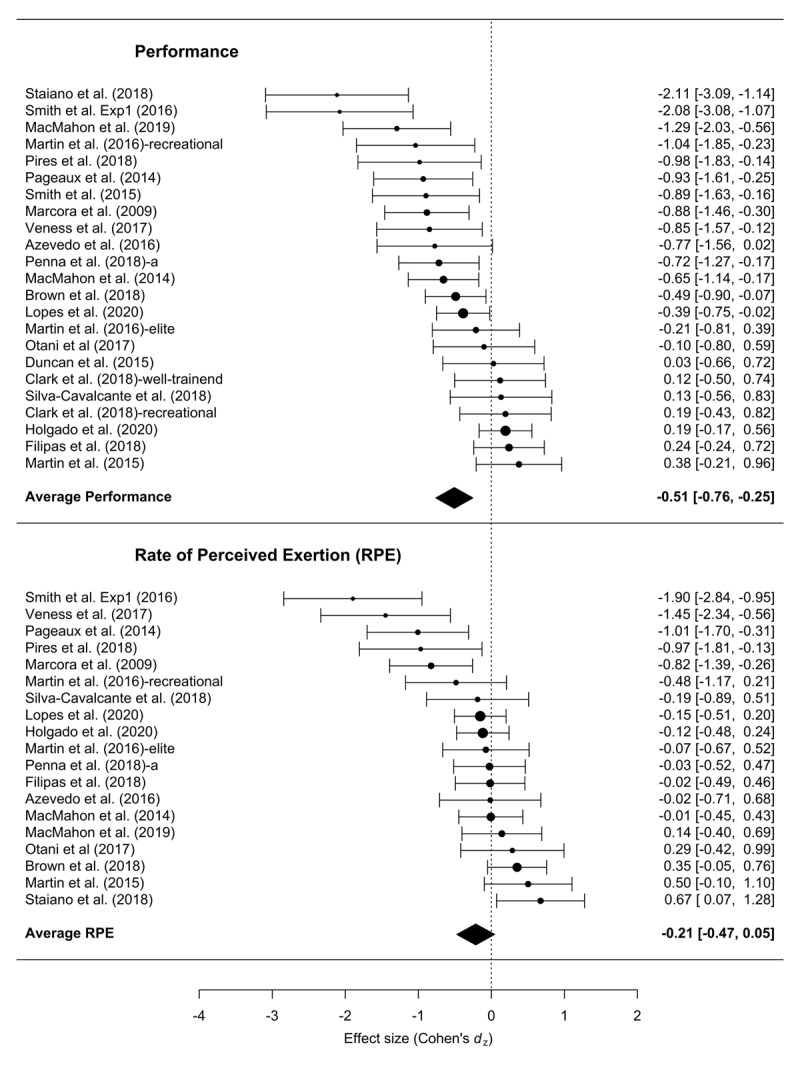
Forest plot of the effect size of mental fatigue on exercise performance and RPE.

In total, we were able to compute 23 statistically independent *d*_z_ scores for endurance performance measures. The results of the overall meta-analysis are summarized in Figure 2. Across all studies, the mean effect size was –0.50, with 95% CI [–0.76, –0.25]. The meta-analysis also revealed a statistically significant amount of heterogeneity across effect sizes, *I*^2^ = 75.4, *Q*(22) = 78.89, *p* < .001. The analysis including the four non-endurance exercise studies yielded a slightly smaller mean effect size of –0.44, with 95% CI [–0.67, –0.20]. The meta-analysis also revealed a statistically significant amount of heterogeneity across effect sizes, *I*^2^ = 76.64, *Q*(26) = 95.94, *p* < .001.

From the 23 studies reporting RPE, the analysis of the 19 testing endurance tasks yielded a mean effect size of –0.21, 95% CI [–0.47, 0.04]. The amount of heterogeneity was also statistically significant, *I*^2^ = 75.18, *Q*(22) = 58.32, *p* < .001. Adding the four non-endurance exercises increased the effect size to –0.27, 95% CI [–0.50, –0.04], with significant heterogeneity, *I*^2^ = 72.56, *Q*(22) = 67.64, *p* < .001.

### Moderator and Sub-Group Analyses

To compare the relative sizes of the effects obtained with objective measures of performance and RPE, we entered the 23 effect sizes of performance and the 19 effect sizes of RPE (from the endurance performance studies) into a single multilevel meta-analysis with the type of outcome (exercise performance vs. RPE) as a categorical moderator, adding a random intercept at the sample level to account for statistical dependencies between effect sizes. The moderator test revealed a significant difference between both types of outcomes, *Q*_M_(1) = 6.41, *p* = .01. As can be seen in the analyses reported above, overall, mental fatigue seemed to have a larger impact on exercise performance measures than on RPE.

The remaining moderator tests were conducted solely on performance measures. Effect sizes were not significantly moderated by the type of participants (recreational vs. well-trained vs. elite), *Q*_M_(2) = 4.04, *p* = .13; recreational participants: *d*_z_ = –0.67, 95% CI [–1.05, –0.29], well-trained participants: *d*_z_ = –0.16, 95% CI [–0.51, 0.17]; and elite participants: *d*_z_ = –0.80, 95% CI [–1.56, –0.5]. Similarly, effect sizes were not moderated by the type of exercise, *Q*_M_(2) = 3.46, *p* = .177, externally-paced exercises, *d*_z_ = –0.69, 95% CI [–1.15, –0.23], self-paced exercise, *d*_z_ = –0.5, 95% CI [–0.82, –0.18], maximal effort exercises, *d*_z_ = 0.23, 95% CI [–0.21, 0.67]. Moreover, effect sizes were not significantly moderated by the length of the fatigue-induction task: *Q*_M_(1) = 0.25, *p* = .718; 30–60 mins, *d*_z_ = –0.62, 95% CI [–0.96, –0.28], and >60 mins, *d*_z_ = –0.30, 95% CI [– 0.67, 0.07]; or the type of cognitive control task: *Q*_M_(1) = 2.10, *p* = .14, less demanding cognitive control task, *d*_z_ = –1.09, 95% CI [–1.59, –0.59], and neutral, *d*_z_ = –0.45, 95% CI [–0.71, –0.18]. Similarly, effect sizes were not significantly larger for studies demonstrating significant evidence of mental fatigue: *Q*_M_(2) = 4.22, *p* = .12, significant evidence of mental fatigue, *d*_z_ = –0.67, 95% CI [–1.01, –0.33]; no significant evidence of mental fatigue, *d*_z_ = –0.41, 95% CI [–0.88, 0.04] and non-reported, *d*_z_ = –0.66, 95% CI [–0.97, –0.35]. Finally, the analysis of motivation was not performed because the intervention affected motivation only in one study. Adding the four non-endurance exercise studies did not change any of these results.

### Analysis of Bias

**Figure 3:**
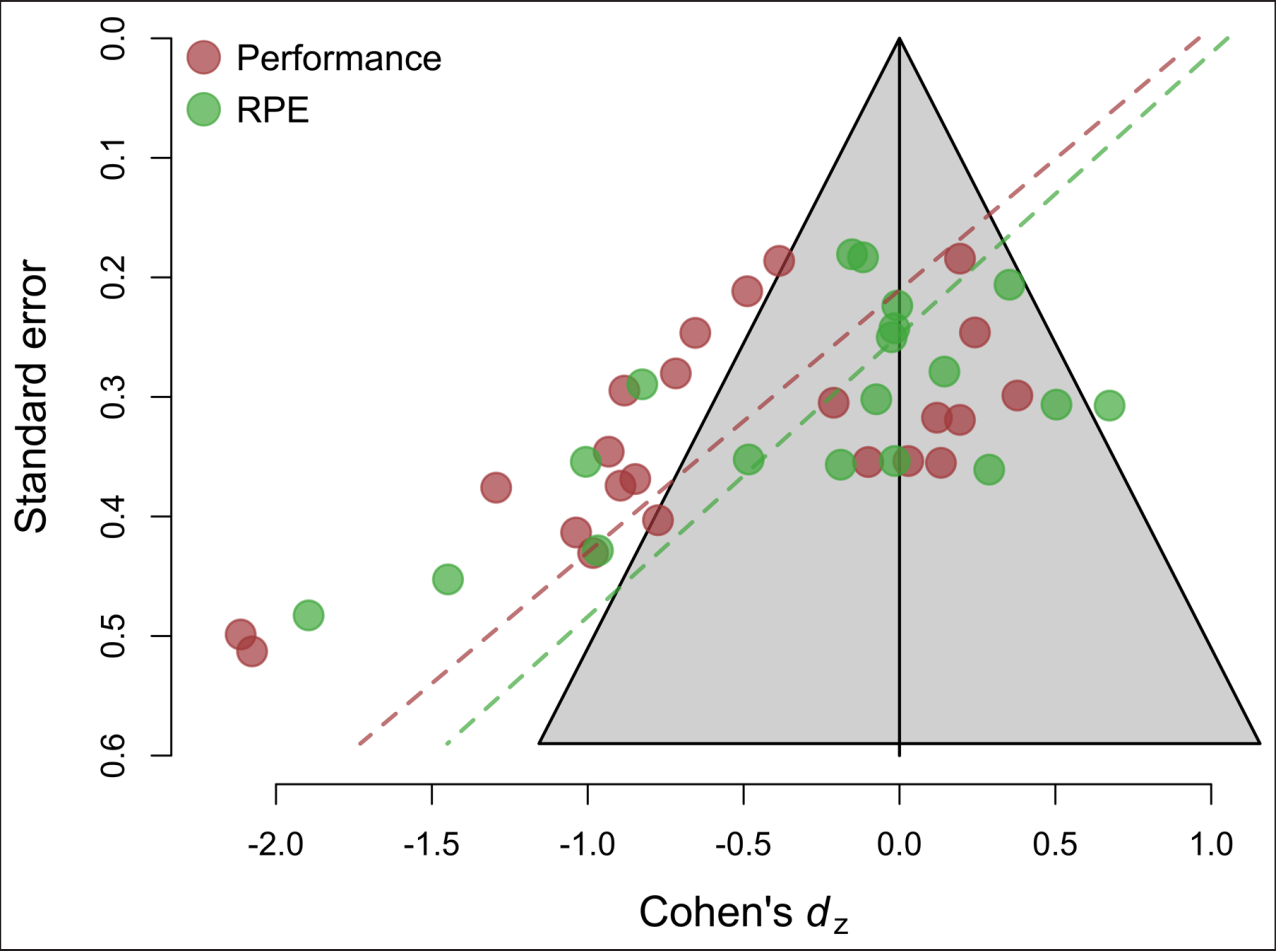
Funnel plot of Cohen’s *d*_z_ effect size versus study standard error.

The funnel plot of effect sizes analyzed depicted in Figure 3 is highly asymmetric for both types of outcomes (performance and RPE), with studies with smaller sample sizes and a higher standard error reporting substantially larger effect sizes. Egger’s test for funnel plot asymmetry was significant for (endurance) performance, *b*_1_ = –4.55, *SE_b_* = 1.3, *z* = 3.50, *p* < .001. This suggests that the distribution of effect sizes might be biased by the selective publication of studies (or analyses) with statistically significant results, and that the meta-analytic average reported above is likely to overestimate the true effects of mental fatigue on these outcomes. Moreover, the intercept of Egger’s test is significantly positive *b*_0_ = 0.96, *SE_b_* = 0.42, *z* = –2.56, *p* = .02, a result that is commonly observed in case of extreme bias (Carter, Schönbrodt, Gervais, & Hilgard, 2019). To obtain an estimate of the likely size of the effect in the absence of publication bias, we fitted a 3-parameter selection model using the weightr package for R (Vevea & Hedges, 1995). Assuming publication bias improved the fit of the model significantly, χ^2^(1) = 7.25, *p* = .007, and returned a non-significant bias-corrected mean effect of –0.08, 95% CI [–0.40, 0.23]. Adding the four non-endurance exercise studies to the sample did not change these results. Egger’s test for funnel plot asymmetry remained significant *b*_1_ = –4.82, *SE_b_* = 1.19, *z* = 4.02, *p* < .001, and the intercept of Egger’s test was also significant and positive *b*_0_ = 1.07, *SE_b_* = 0.38, *z* = –2.80, *p* = .005. The 3-parameter selection model still fitted the data better than the standard random-effects model, χ^2^(1) = 7.33, *p* = .006, and returned a non-significant bias-corrected mean effect of –0.10, 95% CI [–0.31, 0.10].

Likewise, for RPE, Egger’s regression test revealed significant evidence of funnel plot asymmetry, *b*_1_ = –4.24, *SE_b_* = 1.38, *z* = 3.07, *p* = .002, suggesting, again, that the meta-analytic average is likely to be biased by the selective publication of significant results. The intercept of Egger’s regression test was also significantly positive, *b*_0_ = 1.05, *SE_b_* = 0.42, *z* = –2.49, *p* < .012. The bias-corrected average provided by the 3-parameter selection model was small, *d*_z_ = –0.13, and non-significantly different from zero, 95% CI [–0.61, 0.34], although in this case, the model assuming publication bias did not perform significantly better than the standard random-effects model, χ^2^(1) = 0.10, *p* = .74. Likewise, in the analysis including the four non-endurance exercise studies Egger’s test was significant *b*_1_ = –4.26, *SE_b_* = 1.18, *z* = 3.59, *p* < .001, and the intercept of Egger’s regression test was also significant and positive, *b*_0_ = 1.01, *SE_b_* = 0.36, *z* = –2.78, *p* < .005. Similarly, the bias-corrected average provided by the 3-parameter selection model was small, *d*_z_ = –0.15, and non-significantly different from zero, 95% CI [–0.54, 0.24], although again the selection model did not perform significantly better than the standard random-effects model, χ^2^(1) = 0.42, *p* = .516.

## Discussion

The purpose of this systematic review and bias-sensitive meta-analysis was to assess if mental fatigue induced by a long and demanding cognitive task performed before a physical exercise would have a negative influence on performance and RPE. Overall, the analysis seemingly revealed a significant effect of mental fatigue on exercise performance (*d*_z_ = –0.5; *d*_z_= –0.44, including the four non-endurance exercise studies), but supplementary analyses suggested that this estimate was substantially smaller after correcting for selective reporting using the three-parameter selection model (*d*_z_ = –0.08; –0.11, including the four non-endurance exercise studies). Our analyses are in consonance with previous findings (McMorris et al., 2018) and suggest that, once the effects are corrected for publication bias, the resulting average might not be significantly different from zero. Likewise, mental fatigue had a negative influence on RPE (*d*_z_ = –0.21; –0.27), but again the results are probably biased and perhaps not significant once corrected for bias (*d*_z_ = –0.13; –0.15). In sum, our results do not provide strong support for the hypothesis that mental fatigue has a negative influence on exercise performance or RPE. For the sake of fairness, however, the implications of our results should be limited to endurance exercise (although the outcome of the analyses did not change with the inclusion of the four experiments that relied on other types of exercise). Moreover, the conclusions should be also limited to studies with cognitive tasks longer than 30 minutes.

**Figure 4:**
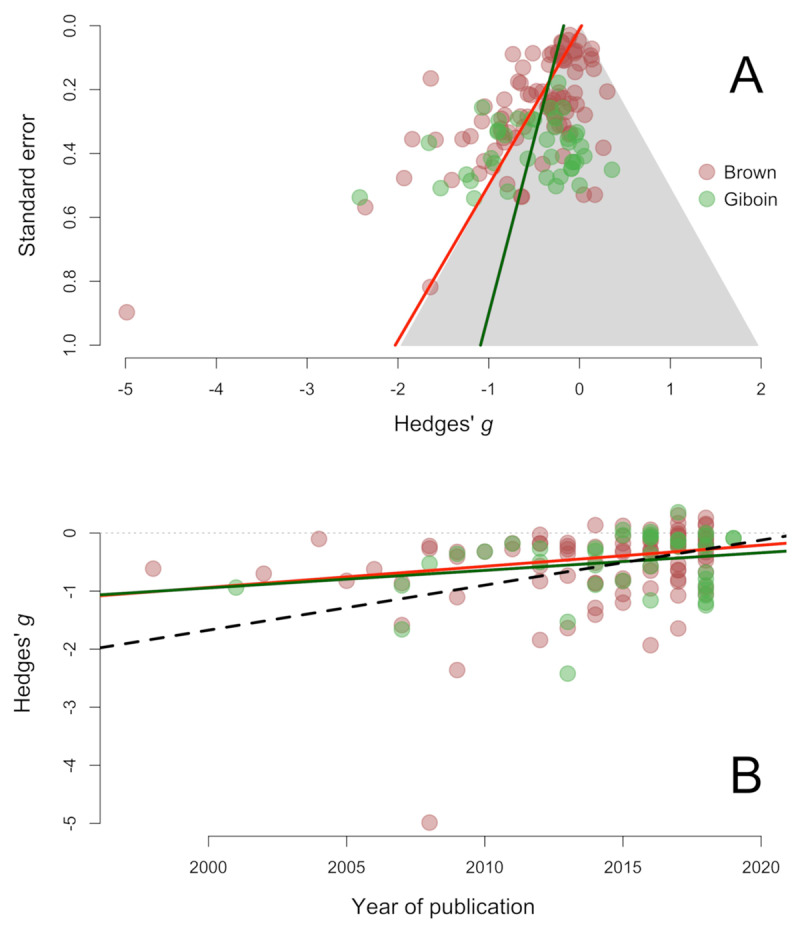
**Panel A:** Funnel plot of the effect sizes meta-analyzed by Brown et al. (2019) and Giboin and Wolff (2019). **Panel B:** Distribution of effect sizes in the same two meta-analyses across time.

These conclusions seem to stand in contrast with some previous meta-analyses conducted in this domain (Brown et al., 2019; Giboin & Wolff, 2019). Brown et al. (2019) performed a meta-analysis based on a similar literature search strategy, except for the fact that their inclusion criteria also considered tasks shorter than 30 mins. The authors concluded that the effects of mental fatigue were robust. However, there are reasons to suspect that their meta-analysis underestimated the potential impact of publication bias. The authors used fail-safe *N* to conclude that the amount of bias observed in this literature is likely to be inconsequential. However, fail-safe *N* is an outdated method that most experts in meta-analysis recommend avoiding. In fact, the Cochrane Handbook explicitly discourages the use of this method (Becker, 2005). Egger’s test, in contrast, shows clear evidence of funnel plot asymmetry. As can be seen in Figure 4A, the best-fitting meta-regression of effect sizes on standard errors overlaps almost perfectly with the border of the grey contour, representing the area of non-significance. Funnel plot asymmetry can arise for reasons other than publication bias, but the striking coincidence between the border of significance and Egger’s regression provides compelling evidence for the hypothesis that this asymmetry is at least partly due to bias. Figure 4A also depicts the effect sizes meta-analyzed by Giboin and Wolff (2019) with the best-fitting Egger’s regression. The evidence of asymmetry is weaker in this case and non-significant. But, as can be seen in the figure, the overall trend is relatively consistent with the asymmetry found in our meta-analysis and in Brown et al. (2019).

Funnel plot asymmetry is not the only piece of evidence suggesting that publication bias might be affecting this literature.^3^ In Figure 4B, we plot the effect sizes meta-analyzed by Brown et al. (2019) and Giboin and Wolff (2019) against their year of publication. In general, the reported effect sizes seem to become closer to zero over the years. This trend is significant in the dataset analyzed by Brown et al. (2019), *b*_1_ = 0.04, *SE*_b_ = 0.01, *z* = –3.11, *p* = .002, and non-significant for Giboin and Wolff, *b*_1_ = 0.03, *SE*_b_ = 0.02, *z* = –1.61, *p* = .107. Note, however, that this does not mean that both patterns of results are in disagreement: in fact, the two meta-regressions overlap almost perfectly. Our own data provides converging evidence for this effect. A multi-level random-effects model predicting both performance and RPE outcomes from year of publication revealed a downward trend (represented by the dotted line in Figure 4B), which approached but failed to reach statistical significance, *b*_1_ = 0.08, *SE*_b_ = 0.04, *z* = –1.89, *p* = .059. This type of decline in effect sizes is observed in many areas of research (Jennions & Møller, 2002; Simmons, Tomkins, Kotiaho, & Hunt, 1999; Vadillo, 2019) and it is usually attributed to the fact that the results of the earliest studies are sometimes inflated by publication or other reporting biases and eventually effect sizes converge to more modest but realistic estimates (Schooler, 2011).

Based on the PEDro quality scores, we might conclude that the results obtained in this review were not influenced by poor methodological design, but the PEDro scale is not without limitations either. Even if the scale considers several items related to the sample of each study (e.g., allocation, blinding), the scale does not take into account how sample sizes were planned. Together with the publication bias aforementioned, our results suggest that the size of the samples tested in these studies could be a cause for concern. For a within-subjects study with two conditions (mental fatigue vs. control), at least 34 participants would be needed to reach 0.8 power to detect an effect of *d*_z_ = –0.50 in a two tailed test with an alpha of .05. For the bias-corrected estimate of *d*_z_ = –0.08, 1,229 participants would be needed to reach the same power. In contrast, the average sample size of the studies included in this meta-analysis was of only 13 participants, which only provided .80 power assuming an (implausible) effect size of *d*_z_ = –0.84. This suggests that most published studies in this literature are underpowered to detect the range of effects that one would expect to find under realistic conditions (Button et al., 2013). It is also important to determine whether such small effects would be relevant to real-world sport performance. Given the current scenario we propose, as an alternative, to conduct multi-lab replications to test larger samples.

Absence of evidence of an effect is not the same as evidence of the absence of an effect. So, despite the fact that the results of the bias-sensitive analysis point to a close-to-null effect, it is still possible that mental fatigue hinders exercise performance. For instance, although, some authors have suggested that there might be a trade-off between performance and RPE, that is, that performance may decline to keep RPE at an optimal level (Staiano, Bosio, de Morree, et al., 2018), it is also plausible that other motivational processes affect performance (Inzlicht, Shenhav, & Olivola, 2018). Exerting a prior mental effort might induce a motivational shift towards more rewarding tasks. If so, athletes would select to exert a lower physical effort, since exercise would represent a costly rather than a rewarding activity (Bieleke & Wolff, In press). However, among the included studies, only one revealed that motivation toward exercise changed compared to the baseline measure.

The absence of clear evidence of these effects might be due to the high interindividual variability in the participants’ state of mental fatigue after the cognitive task. As an alternative for future studies, we propose that the amount of cognitive load should be individualized to guarantee that every participant reaches comparable levels of mental fatigue. For example, the study by O’Keeffe et al., (2020) showed that an individualized cognitive load in a dual-task was more effective at inducing a high level of mental fatigue than the AX-CPT. This could be one of the factors explaining that some of the studies in the present meta-analysis failed to detect an (overall) subjective feeling of mental fatigue. For the purpose of comparison, we propose using two levels of (individualized) mental load of the same cognitive task. In addition, though little is known in this regard, mental fatigue accrued over consecutive days might have a stronger influence on exercise performance than the typical one-session manipulation used to date (Russell, Jenkins, Smith, Halson, & Kelly, 2019). Researchers could use the “rating of fatigue scale” (Micklewright, St Clair Gibson, Gladwell, & Al Salman, 2017) together with additional objective indexes of fatigue (e.g., brain activity; Tran, Craig, Craig, Chai, & Nguyen, 2020; Wang et al., 2016), to assess the multifaceted nature of fatigue (Enoka & Duchateau, 2016).

Last, but not least, assuming that cognitive (executive) processing is relevant for exercise regulation and that mental fatigue has a negative effect on endurance performance, it is likely that experts or high-fit participants might have reached higher levels of automaticity and that the cognitive demands for performing exercise might be reduced in them. Although our moderator analyses did not show any significant evidence, mental fatigue induced in a lab-setting can be expected to have a lower influence on exercise performance in fitter participants (Holgado & Sanabria, 2020; Russell et al., 2019). However, classifying the fit level of participants is still a daunting challenge, given the variety of descriptions and classifications of samples across studies (De Pauw et al., 2013). Nevertheless, as mentioned above, the first step should be to guarantee that all participants reach a similar state of mental fatigue, ideally assessed both subjectively and objectively. Finally, self-paced, externally-paced, or maximal effort exercise modalities may involve different cognitive processes and, consequently, might be affected differently by mental fatigue (Marino, 2012).

The present meta-analysis is not without limitations. Contrary to previous meta-analyses, we did not include studies with cognitive manipulations lasting less than 30 minutes, so we could be ignoring a meaningful part of the literature investigating this phenomenon. Note, in any case, that our results seem to converge with those of other meta-analyses, which included shorter interventions (Brown et al., 2019; Giboin & Wolff, 2019; McMorris et al., 2018). In addition, although we searched for grey literature, it is likely that we missed unpublished studies, which might be considered as a limitation of our meta-analysis.

## Conclusion

The results of the current meta-analysis raise concerns about the nature and strength of the effects of mental fatigue on exercise. In light of the potential presence of reporting biases in this literature, it is difficult to draw any firm conclusion about the link between mental fatigue and exercise performance or perceived exertion. Testing larger samples, perhaps by means of multi-lab replications, might help to improve the evidential value of this literature, given that the average sample among the studies we reviewed was only 13 participants. To ameliorate the impact of publication bias in future meta-analysis, we recommend that researchers pre-register their protocols and also that they share the data and materials in public repositories. All the data and scripts used here are publicly available at https://osf.io/s5tz6/.

## Data Accessibility Statement

Data and code for the meta-analysis can be found here: https://osf.io/s5tz6/.

## Additional File

The additional file for this article can be found as follows:

10.5334/joc.126.s1 Table 1. Studies examining the effects of mental fatigue on exercise performance.

## Footnotes

Some authors have referred this state as a cognitive fatigue rather than mental fatigue, but, as in previous reviews, we will not make differences between terms “mental fatigue” and “cognitive fatigue”, and we will use mental fatigue consistently (Pageaux & Lepers, 2018; Van Cutsem, Marcora, et al., 2017).

Self-paced exercise refers to a physical activity in which the athlete has to regulate the effort throughout the test, whereas in an externally-paced exercise, intensity is fixed and athletes cannot change the pacing.

Some peculiarities of the meta-analyses conducted by Brown et al. (2019) and Giboin and Wolff (2019) prevent the use of the 3-parameter selection model to test for publication bias. Both meta-analyses collated data from studies with within-participants designs and between-groups designs. To do this, meta-analysts cannot use *d*_z_ scores as an effect size estimate for within-participants designs, because those scores are not directly comparable to the Cohen’s *d* estimates used for between-groups designs. The usual approach to improve the comparability of effect sizes is to compute an alternative type of Cohen’s *d* for within-participants designs (Morris & DeShon, 2002). An unfortunate consequence of this is that the resulting *d* scores for within-participants designs and their 95% confidence intervals may no longer agree with the statistical significance of that effect in the original analyses reported in the primary studies. That is, a contrast that was originally reported as statistically significant may be translated to an effect size estimate with a confidence interval that includes zero, or vice versa. The 3-parameter selection model assumes that effect sizes may have a lower probability of being published if their confidence intervals include zero. But of course, this logic breaks down if the significance of the effect size estimates is detached from the significance of the corresponding analysis in the original paper where the study was reported.

## References

[1] Audiffren, M., & André, N. (2015). The strength model of self-control revisited: Linking acute and chronic effects of exercise on executive functions. Journal of Sport and Health Science, 4(1), 30–46. DOI: 10.1016/j.jshs.2014.09.002

[2] Azevedo, R., Silva-Cavalcante, M. D., Gualano, B., Lima-Silva, A. E., & Bertuzzi, R. (2016). Effects of caffeine ingestion on endurance performance in mentally fatigued individuals European Journal of Applied Physiology, 116(11–12), 2293–2303. Scopus DOI: 10.1007/s00421-016-3483-y 27695980

[3] Badin, O. O., Smith, M. R., Conte, D., & Coutts, A. J. (2016). Mental fatigue: Impairment of technical performance in small-sided soccer games International Journal of Sports Physiology and Performance, 11(8), 1100–1105. Scopus DOI: 10.1123/ijspp.2015-0710 27003948

[4] Baron, B., Moullan, F., Deruelle, F., & Noakes, T. D. (2011). The role of emotions on pacing strategies and performance in middle and long duration sport events. British Journal of Sports Medicine, 45(6), 511–517. DOI: 10.1136/bjsm.2009.059964 19553226

[5] Becker, J. (2005). Failsafe N or File-Drawer Number In H. Rothstein, M. Sutton & M. Borenstein (Eds.), Publication Bias in Meta-Analysis: Prevention, Assessment and Adjustments. Wiley Retrieved from https://www.wiley.com/en-es/Publication+Bias+in+Meta+Analysis%3A+Prevention%2C+Assessment+and+Adjustments-p-9780470870143 DOI: 10.1002/0470870168.ch7

[6] Bieleke, M., & Wolff, W. (In press). Editorial: The self-regulation of human performance. Performance Enhancement & Health, 100166.

[7] Brown, D. M. Y., & Bray, S. R. (2019). Effects of Mental Fatigue on Exercise Intentions and Behavior. Annals of Behavioral Medicine, 53(5), 405–414. DOI: 10.1093/abm/kay052 29985969

[8] Brown, D. M. Y., Graham, J. D., Innes, K. I., Harris, S., Flemington, A., & Bray, S. R. (2019). Effects of Prior Cognitive Exertion on Physical Performance: A Systematic Review and Meta-analysis. Sports Medicine. DOI: 10.1007/s40279-019-01204-8 31873926

[9] Brownsberger, J., Edwards, A., Crowther, R., & Cottrell, D. (2013). Impact of mental fatigue on self-paced exercise. International Journal of Sports Medicine, 34(12), 1029–1036. DOI: 10.1055/s-0033-1343402 23771830

[10] Button, K. S., Ioannidis, J. P. A., Mokrysz, C., Nosek, B. A., Flint, J., Robinson, E. S. J., & Munafò, M. R. (2013). Power failure: Why small sample size undermines the reliability of neuroscience. Nature Reviews Neuroscience, 14(5), 365–376. DOI: 10.1038/nrn3475 23571845

[11] Carter, E. C., Schönbrodt, F. D., Gervais, W. M., & Hilgard, J. (2019). Correcting for Bias in Psychology: A Comparison of Meta-Analytic Methods. Advances in Methods and Practices in Psychological Science, 2(2), 115–144. DOI: 10.1177/2515245919847196

[12] Clark, I. E., Goulding, R. P., DiMenna, F. J., Bailey, S. J., Jones, M. I., Fulford, J., … Vanhatalo, A. (2019). Time-trial performance is not impaired in either competitive athletes or untrained individuals following a prolonged cognitive task European Journal of Applied Physiology, 119(1), 149–161. Scopus DOI: 10.1007/s00421-018-4009-6 30443808 PMC6342897

[13] De Pauw, K., Roelands, B., Cheung, S. S., de Geus, B., Rietjens, G., & Meeusen, R. (2013). Guidelines to classify subject groups in sport-science research. International Journal of Sports Physiology and Performance, 8(2), 111–122. DOI: 10.1123/ijspp.8.2.111 23428482

[14] Duncan, M. J., Fowler, N., George, O., Joyce, S., & Hankey, J. (2015). Mental fatigue negatively influences manual dexterity and anticipation timing but not repeated high-intensity exercise performance in trained adults Research in Sports Medicine, 23(1), 1–13. Scopus DOI: 10.1080/15438627.2014.975811 25630242

[15] Enoka, R. M., & Duchateau, J. (2016). Translating Fatigue to Human Performance. Medicine and Science in Sports and Exercise, 48(11), 2228–2238. DOI: 10.1249/MSS.0000000000000929 27015386 PMC5035715

[16] Filipas, L., Mottola, F., Tagliabue, G., & La Torre, A. (2018). The effect of mentally demanding cognitive tasks on rowing performance in young athletes. Psychology of Sport and Exercise, 39, 52–62. DOI: 10.1016/j.psychsport.2018.08.002

[17] Giboin, L.-S., & Wolff, W. (2019). The effect of ego depletion or mental fatigue on subsequent physical endurance performance: A meta-analysis. Performance Enhancement & Health, 7(1), 100150 DOI: 10.1016/j.peh.2019.100150

[18] Head, J. R., Tenan, M. S., Tweedell, A. J., Price, T. F., LaFiandra, M. E., & Helton, W. S. (2016). Cognitive fatigue influences time-on-task during bodyweight resistance training exercise Frontiers in Physiology, 7(SEP). Scopus DOI: 10.3389/fphys.2016.00373 PMC5007724 27635122

[19] Holgado, D., & Sanabria, D. (2020). Does self-paced exercise depend on executive processing? A narrative review of the current evidence. International Review of Sport and Exercise Psychology. DOI: 10.1080/1750984X.2020.1774915

[20] Holgado, D., Troya, E., Perales, J. C., Vadillo, M., & Sanabria, D. (2020). Does mental fatigue impair physical performance? A replication study. European Journal of Sport Science. DOI: 10.1080/17461391.2020.1781265 32519588

[21] Inzlicht, M., Shenhav, A., & Olivola, C. Y. (2018). The Effort Paradox: Effort Is Both Costly and Valued. Trends in Cognitive Sciences, 22(4), 337–349. DOI: 10.1016/j.tics.2018.01.007 29477776 PMC6172040

[22] Ishii, A., Tanaka, M., & Watanabe, Y. (2014). Neural mechanisms of mental fatigue. Reviews in the Neurosciences, 25(4), 469–479. DOI: 10.1515/revneuro-2014-0028 24926625

[23] Jennions, M. D., & Møller, A. P. (2002). Relationships fade with time: A meta-analysis of temporal trends in publication in ecology and evolution. Proceedings of the Royal Society of London. Series B: Biological Sciences, 269(1486), 43–48. DOI: 10.1098/rspb.2001.1832 11788035 PMC1690867

[24] Lopes, T. R., Oliveira, D. M., Simurro, P. B., Akiba, H. T., Nakamura, F. Y., Okano, A. H., … Silva, B. M. (2020). No Sex Difference in Mental Fatigue Effect on High-Level Runners’ Aerobic Performance. Medicine and Science in Sports and Exercise. DOI: 10.1249/MSS.0000000000002346 32251253

[25] MacMahon, C., Hawkins, Z., & Schücker, L. (2019). Beep Test Performance Is Influenced by 30 Minutes of Cognitive Work. Medicine and Science in Sports and Exercise. DOI: 10.1249/MSS.0000000000001982 PMC6727939 30913161

[26] MacMahon, C., Schücker, L., Hagemann, N., & Strauss, B. (2014). Cognitive fatigue effects on physical performance during running. Journal of Sport & Exercise Psychology, 36(4), 375–381. DOI: 10.1123/jsep.2013-0249 25226606

[27] Marcora, S., Staiano, W., & Manning, V. (2009). Mental fatigue impairs physical performance in humans. Journal of Applied Physiology, 106(3), 857–864. DOI: 10.1152/japplphysiol.91324.2008 19131473

[28] Marino, F. E. (2012). The limitations of the constant load and self-paced exercise models of exercise physiology. Comparative Exercise Physiology, 8(1), 3–9. DOI: 10.3920/CEP11012

[29] Martin, K., Staiano, W., Menaspà, P., Hennessey, T., Marcora, S., Keegan, R., … Rattray, B. (2016). Superior Inhibitory Control and Resistance to Mental Fatigue in Professional Road Cyclists. PLOS ONE, 11(7), e0159907 DOI: 10.1371/journal.pone.0159907 27441380 PMC4956323

[30] Martin, K., Thompson, K. G., Keegan, R., Ball, N., & Rattray, B. (2015). Mental fatigue does not affect maximal anaerobic exercise performance European Journal of Applied Physiology, 115(4), 715–725. Scopus DOI: 10.1007/s00421-014-3052-1 25425259

[31] McMorris, T., Barwood, M., Hale, B., Dicks, M., & Corbett, J. (2018). Cognitive fatigue effects on physical performance: A systematic review and meta-analysis. Physiology & Behavior, 188, 103–107. DOI: 10.1016/j.physbeh.2018.01.029 29408319

[32] Micklewright, D., St Clair Gibson, A., Gladwell, V., & Al Salman, A. (2017). Development and Validity of the Rating-of-Fatigue Scale. Sports Medicine. DOI: 10.1007/s40279-017-0711-5 PMC5633636 28283993

[33] Moher, D., Liberati, A., Tetzlaff, J., & Altman, D. G. (2009). Preferred reporting items for systematic reviews and meta-analyses: The PRISMA statement. BMJ (Clinical Research Ed.), 339, b2535 DOI: 10.1136/bmj.b2535 PMC2714657 19622551

[34] Morris, S. B., & DeShon, R. P. (2002). Combining effect size estimates in meta-analysis with repeated measures and independent-groups designs. Psychological Methods, 7(1), 105–125. DOI: 10.1037/1082-989X.7.1.105 11928886

[35] O’Keeffe, K., Hodder, S., & Lloyd, A. (2020). A comparison of methods used for inducing mental fatigue in performance research: Individualised, dual-task and short duration cognitive tests are most effective. Ergonomics, 63(1), 1–12. DOI: 10.1080/00140139.2019.1687940 31680632

[36] Otani, H., Kaya, M., Tamaki, A., & Watson, P. (2017). Separate and combined effects of exposure to heat stress and mental fatigue on endurance exercise capacity in the heat European Journal of Applied Physiology, 117(1), 119–129. Scopus DOI: 10.1007/s00421-016-3504-x 27864637

[37] Ouzzani, M., Hammady, H., Fedorowicz, Z., & Elmagarmid, A. (2016). Rayyan—A web and mobile app for systematic reviews. Systematic Reviews, 5(1). DOI: 10.1186/s13643-016-0384-4 PMC5139140 27919275

[38] Pageaux, B. (2014). The psychobiological model of endurance performance: An effort-based decision-making theory to explain self-paced endurance performance. Sports Medicine (Auckland, N.Z.), 1319–1320. DOI: 10.1007/s40279-014-0198-2 24809249

[39] Pageaux, B., & Lepers, R. (2018). Chapter 16—The effects of mental fatigue on sport-related performance In S. Marcora & M. Sarkar (Eds.), Progress in Brain Research (pp. 291–315). Elsevier DOI: 10.1016/bs.pbr.2018.10.004 30390836

[40] Pageaux, B., Marcora, S. M., & Lepers, R. (2013). Prolonged mental exertion does not alter neuromuscular function of the knee extensors Medicine and Science in Sports and Exercise, 45(12), 2254–2264. Scopus DOI: 10.1249/MSS.0b013e31829b504a 23698244

[41] Pageaux, B., Lepers, R., Dietz, K. C., & Marcora, S. M. (2014). Response inhibition impairs subsequent self-paced endurance performance. European Journal of Applied Physiology, 114(5), 1095–1105. DOI: 10.1007/s00421-014-2838-5 24531591

[42] Penna, E. M., Filho, E., Campos, B. T., Pires, D. A., Nakamura, F. Y., Mendes, T. T., … Prado, L. S. (2018a). Mental fatigue does not affect heart rate recovery but impairs performance in handball players Revista Brasileira de Medicina Do Esporte, 24(5), 347–351. Scopus DOI: 10.1590/1517-869220182405180483

[43] Penna, E. M., Filho, E., Wanner, S. P., Campos, B. T., Quinan, G. R., Mendes, T. T., … Prado, L. S. (2018b). Mental Fatigue Impairs Physical Performance in Young Swimmers. Pediatric Exercise Science, 30(2), 208–215. DOI: 10.1123/pes.2017-0128 29276857

[44] Pires, F. O., Silva-Júnior, F. L., Brietzke, C., Franco-Alvarenga, P. E., Pinheiro, F. A., de França, N. M., … Meireles Santos, T. (2018). Mental Fatigue Alters Cortical Activation and Psychological Responses, Impairing Performance in a Distance-Based Cycling Trial. Frontiers in Physiology, 9 DOI: 10.3389/fphys.2018.00227 PMC5864900 29615923

[45] Proske, U., & Gandevia, S. C. (2012). The Proprioceptive Senses: Their Roles in Signaling Body Shape, Body Position and Movement, and Muscle Force. Physiological Reviews, 92(4), 1651–1697. DOI: 10.1152/physrev.00048.2011 23073629

[46] Roelands, B., De Koning, J., Foster, C., Hettinga, F., & Meeusen, R. (2013). Neurophysiological determinants of theoretical concepts and mechanisms involved in pacing. Sports Medicine, 43(5), 301–311. DOI: 10.1007/s40279-013-0030-4 23456493

[47] Russell, S., Jenkins, D., Smith, M., Halson, S., & Kelly, V. (2019). The application of mental fatigue research to elite team sport performance: New perspectives. Article in Press, Scopus DOI: 10.1016/j.jsams.2018.12.008 30606625

[48] Salam, H., Marcora, S. M., & Hopker, J. G. (2018). The effect of mental fatigue on critical power during cycling exercise European Journal of Applied Physiology, 118(1), 85–92. Scopus DOI: 10.1007/s00421-017-3747-1 29124324 PMC5754415

[49] Schooler, J. (2011). Unpublished results hide the decline effect. Nature, 470(7335), 437 DOI: 10.1038/470437a 21350443

[50] Silva-Cavalcante, M. D., Couto, P. G., Azevedo, R. A., Silva, R. G., Coelho, D. B., Lima-Silva, A. E., & Bertuzzi, R. (2018). Mental fatigue does not alter performance or neuromuscular fatigue development during self-paced exercise in recreationally trained cyclists European Journal of Applied Physiology, 118(11), 2477–2487. Scopus DOI: 10.1007/s00421-018-3974-0 30155760

[51] Simmons, L. W., Tomkins, J. L., Kotiaho, J. S., & Hunt, J. (1999). Fluctuating paradigm. Proceedings of the Royal Society of London. Series B: Biological Sciences, 266(1419), 593–595. DOI: 10.1098/rspb.1999.0677

[52] Slimani, M., Znazen, H., Bragazzi, N. L., Zguira, M. S., & Tod, D. (2018). The Effect of Mental Fatigue on Cognitive and Aerobic Performance in Adolescent Active Endurance Athletes: Insights from a Randomized Counterbalanced, Cross-Over Trial. Journal of Clinical Medicine, 7(12), 510 DOI: 10.3390/jcm7120510 PMC6306934 30513903

[53] Smith, M. R., Coutts, A. J., Merlini, M., Deprez, D., Lenoir, M., & Marcora, S. M. (2016). Mental fatigue impairs soccer-specific physical and technical performance Medicine and Science in Sports and Exercise, 48(2), 267–276. Scopus DOI: 10.1249/MSS.0000000000000762 26312616

[54] Smith, M. R., Marcora, S. M., & Coutts, A. J. (2015). Mental fatigue impairs intermittent running performance Medicine and Science in Sports and Exercise, 47(8), 1682–1690. Scopus DOI: 10.1249/MSS.0000000000000592 25494389

[55] Staiano, W., Bosio, A., de Morree, H. M., Rampinini, E., & Marcora, S. (2018). The cardinal exercise stopper: Muscle fatigue, muscle pain or perception of effort? Progress in Brain Research, 240, 175–200. DOI: 10.1016/bs.pbr.2018.09.012 30390830

[56] Staiano, W., Bosio, A., Piazza, G., Romagnoli, M., & Invernizzi, P. L. (2018). Kayaking performance is altered in mentally fatigued young elite athletes. The Journal of Sports Medicine and Physical Fitness. DOI: 10.23736/S0022-4707.18.09051-5 30317839

[57] Tran, Y., Craig, A., Craig, R., Chai, R., & Nguyen, H. (2020). The influence of mental fatigue on brain activity: Evidence from a systematic review with meta-analyses. Psychophysiology, 57(5), e13554 DOI: 10.1111/psyp.13554 32108954

[58] Vadillo, M. A. (2019). Ego Depletion May Disappear by 2020. Social Psychology, 50(5–6), 282–291. DOI: 10.1027/1864-9335/a000375

[59] Van Cutsem, J., De Pauw, K., Buyse, L., Marcora, S., Meeusen, R., & Roelands, B. (2017). Effects of mental fatigue on endurance performance in the heat Medicine and Science in Sports and Exercise, 49(8), 1677–1687. Scopus DOI: 10.1249/MSS.0000000000001263 28282326

[60] Van Cutsem, J., Marcora, S., Pauw, K. D., Bailey, S., Meeusen, R., & Roelands, B. (2017). The Effects of Mental Fatigue on Physical Performance: A Systematic Review. Sports Medicine, 47(8), 1569–1588. DOI: 10.1007/s40279-016-0672-0 28044281

[61] Veness, D., Patterson, S. D., Jeffries, O., & Waldron, M. (2017). The effects of mental fatigue on cricket-relevant performance among elite players Journal of Sports Sciences, 35(24), 2461–2467. Scopus DOI: 10.1080/02640414.2016.1273540 28092208

[62] Verhagen, A. P., de Vet, H. C., de Bie, R. A., Kessels, A. G., Boers, M., Bouter, L. M., & Knipschild, P. G. (1998). The Delphi list: A criteria list for quality assessment of randomized clinical trials for conducting systematic reviews developed by Delphi consensus. Journal of Clinical Epidemiology, 51(12), 1235–1241. DOI: 10.1016/S0895-4356(98)00131-0 10086815

[63] Verhoeven, K., Van Damme, S., Eccleston, C., Van Ryckeghem, D. M. L., Legrain, V., & Crombez, G. (2011). Distraction from pain and executive functioning: An experimental investigation of the role of inhibition, task switching and working memory. European Journal of Pain (London, England), 15(8), 866–873. DOI: 10.1016/j.ejpain.2011.01.009 21397536

[64] Vevea, J. L., & Hedges, L. V. (1995). A general linear model for estimating effect size in the presence of publication bias. Psychometrika, 60(3), 419–435. DOI: 10.1007/BF02294384

[65] Viechtbauer, W. (2010). Conducting Meta-Analyses in R with the metafor Package. Journal of Statistical Software. DOI: 10.18637/jss.v036.i03

[66] Vrijkotte, S., Meeusen, R., Vandervaeren, C., Buyse, L., Van Cutsem, J., Pattyn, N., & Roelands, B. (2018). Mental fatigue and physical and cognitive performance during a 2-bout exercise test International Journal of Sports Physiology and Performance, 13(4), 510–516. Scopus DOI: 10.1123/ijspp.2016-0797 28952829

[67] Wang, C., Trongnetrpunya, A., Babu, I., Samuel, H., Ding, M., & Kluger, B. M. (2016). Compensatory Neural Activity in Response to Cognitive Fatigue. 36(14), 3919–3924. DOI: 10.1523/JNEUROSCI.3652-15.2016 PMC4821906 27053200

