# Correction: Mental Fatigue Might be Not So Bad for Exercise Performance After All: A Systematic Review and Bias-Sensitive Meta-Analysis

**DOI:** 10.5334/joc.178

**Published:** 2021-07-19

**Authors:** Darías Holgado, Daniel Sanabria, José C. Perales, Miguel A. Vadillo

**Affiliations:** 1 Centro de Investigación, Mente, Cerebro y Comportamiento (CIMCYC), Universidad de Granada, ES; 2 Departamento de Psicología Experimental, Universidad de Granada, ES; 3 Departamento de Psicología Básica, Universidad Autónoma de Madrid, ES

**Keywords:** Cognitive Control, Attention, Face perception, EEG, Emotion and cognition

## Abstract

This article details a correction to: Holgado, D., Sanabria, D., Perales, J. C., & Vadillo, M. A. (2020). Mental Fatigue Might Be Not So Bad for Exercise Performance After All: A Systematic Review and Bias-Sensitive Meta-Analysis. Journal of Cognition, 3(1), 38. DOI: *https://doi.org/10.5334/joc.126*

## Correction

After revising the dataset and analysis for our meta-analysis *“Mental fatigue might be not so bad for exercise performance after all: a systematic review and bias-sensitive meta-analysis” (Holgado et al., 2020)*, we detected some errors in the computation of effect sizes that we would like to correct. None of these corrections makes a meaningful difference in the interpretation of the results, but they do affect the numerical values of the effect size estimates.

The signs of some of the effect sizes included in our meta-analysis were miscoded. The reader should keep in mind that in our meta-analysis a negative sign means that effect size favours the hypothesis of worse performance in the mental fatigue condition or higher perceived effort in the mental fatigue condition. According to this, the following effect sizes should be inverted: performance in Filipas et al. (2018) (from positive to negative); RPE in Martin et al. (2016) (from negative to positive); and RPE in Martin et al. (2015) and in Staiano et al. (2018) (from positive to negative).

After correcting these values and including two additional RPE measures that we missed in the original meta-analysis (Smith et al., 2015 and Duncan et al., 2015), we have repeated all the analyses. As mentioned above, the results do not change substantially with respect to those reported in the published paper, and therefore the conclusions of the manuscript remain unchanged. The corrected estimated effect size and forest plot are reported below.

## Analyses

### Performance

Across all studies, the mean effect size was –0.53 (***Figure 1***), with 95% CI [–0.76, –0.28] (compared to –0.50, with 95% CI [–0.76, –0.25] in the original meta-analysis). The meta-analysis also revealed a statistically significant amount of heterogeneity across effect sizes, *I*^2^ = 72.88, *Q*(22) = 72.53, *p* < .001. The analysis including the four non-endurance exercise studies yielded a slightly smaller mean effect size of –0.49, with 95% CI [–0.70, –0.28] (in the previous version –0.44, with 95% CI [–0.67, –0.20]). The meta-analysis also revealed a statistically significant amount of heterogeneity across effect sizes, *I*^2^ = 70.95, *Q*(26) = 80.91, *p* < .001. Adding the four non-endurance exercise studies to the sample did not change these results.

**Figure 1:**
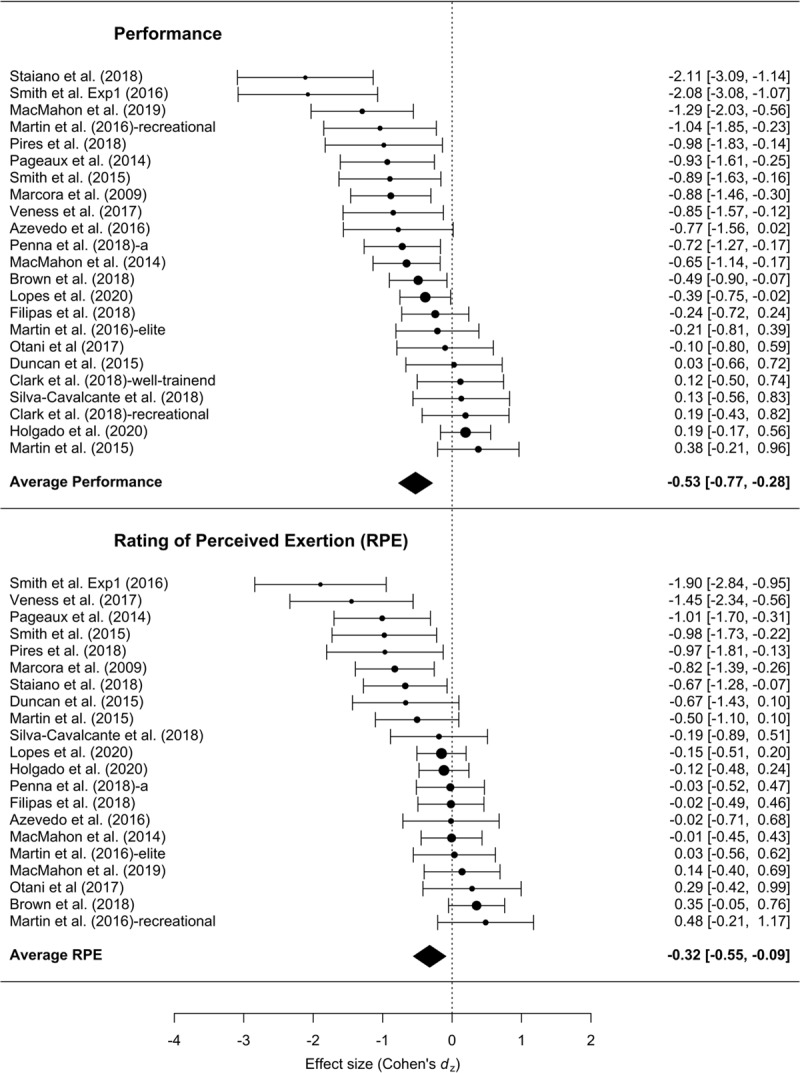
Forest plot of the effect size of mental fatigue on exercise performance and RPE.

### RPE

From the 24 studies reporting RPE, the analysis of the 21 testing endurance tasks yielded a mean effect size of –0.32, 95% CI [–0.55, –0.09] (in the previous version –0.21, 95% CI [–0.47, 0.04]). The amount of heterogeneity was also statistically significant, *I*^2^ = 70.52, *Q*(20) = 59.08, *p* < .001. Adding the non-endurance exercises the effect size slightly changed to –0.32, 95% CI [–0.52, –0.12] (before –0.27, 95% CI [–0.50, –0.04]), with significant heterogeneity, *I*^2^ = 66.13, *Q*(23) = 61.85, *p* < .001.

#### Moderator analysis on Performance for endurance exercise

Effect sizes were not significantly moderated by the type of participants (recreational vs. well-trained vs. elite), *Q_M_*(2) = 3.17, p = .20; recreational participants: *d_z_* = –0.67, 95% CI [–1.05, –0.29], well-trained participants: *d_z_* = –0.24, 95% CI [–0.55, 0.07]; and elite participants: d_z_ = –0.80, 95% CI [–1.56, –0.5]. Similarly, effect sizes were not moderated by the type of exercise, *Q_M_*(2) = 3.83, p = .14, externally-paced exercises, *d_z_* = –0.69, 95% CI [–1.15, –0.23], self-paced exercise, *d_z_* = –0.54, 95% CI [–0.82, –0.25], maximal effort exercises, *d_z_* = 0.23, 95% CI [–0.21, 0.67]. Moreover, effect sizes were not significantly moderated by the length of the fatigue-induction task: *Q_M_*(1) = 1.18, p = .17; 30–60 mins *d_z_* = –0.65, 95% CI [–0.97, –0.33], and. >60 mins, *d_z_* = –0.30, 95% CI [–0.67, 0.07]; or the type of cognitive control task: *Q_M_*(1) = 2.15, p = .14, less demanding cognitive control task, *d_z_* = –1.09, 95% CI [–1.59, –0.59], and neutral, *d_z_* = –0.47, 95% CI [–0.72, –0.22], Similarly, effect sizes were not significantly larger for studies demonstrating significant evidence of mental fatigue: *Q_M_*(2) = 5.21, p = .07, significant evidence of mental fatigue, *d_z_* = –0.69, 95% CI [–1.01, –0.38]; no significant evidence of mental fatigue, *d_z_* = –0.41, 95% CI [–0.88, 0.04] and non reported, *d_z_* = 0.11, 95% CI [–0.25, 0.49].

#### Moderator analysis on the RPE measures for endurance exercise

Effect sizes were not significantly moderated by the type of participants (recreational vs. well-trained vs. elite), *Q_M_*(2) = 0.34, p = .84; recreational participants: *d_z_* = –0.30, 95% CI [–0.74, 0.13], well-trained participants: *d_z_* = –0.24, 95% CI [–0.48, 0.0]; and elite participants: *d_z_* = –0.48, 95% CI [–1.05, 0.08]. Similarly, effect sizes were not moderated by the type of exercise, *Q_M_*(2) = 0.87, p = .64, externally-paced exercises, *d_z_* = –0.43, 95% CI [–0.97, 0.05], self-paced exercise, *d_z_* = –0.21, 95% CI [–0.51, 0.07], maximal effort exercises, *d_z_* = –0.56, 95% CI [–1.03, –0.09]. Moreover, effect sizes were not significantly moderated by the length of the fatigue-induction task: *Q_M_*(1) = 0.07, p = .77; 30–60 mins *d*_z_ = –0.37, 95% CI [–0.73, –0.01], and. >60 mins, *d_z_* = –0.27, 95% CI [–0.54, –0.003]; or the type of cognitive control task: *Q_M_*(1) = 0.02, p = .87, less demanding cognitive control task, *d_z_* = –0.41, 95% CI [–1.53, 0.71], and neutral, *d_z_* = –0.31, 95% CI [–0.55, –0.07]. Similarly, effect sizes were not significantly larger for studies demonstrating significant evidence of mental fatigue: *Q_M_*(2) = 0.33, p = .84, significant evidence of mental fatigue, *d_z_* = –0.33, 95% CI [–0.64, –0.02]; no significant evidence of mental fatigue, *d_z_* = –0.26, 95% CI [–0.63, 0.1] and non reported, *d_z_* = –0.66, 95% CI [–1.43, 0.09].

#### Analysis of Publication Bias

Egger’s test for funnel plot asymmetry (***Figure 2***) was significant for (endurance) performance, *b_1_* = –4.25, *SE_b_* = 1.26, *z* = –3.37, *p* < .001. This suggests that the distribution of effect sizes might be biased by the selective publication of studies (or analyses) with statistically significant results, and that the meta-analytic average reported above is likely to overestimate the true effects of mental fatigue on these outcomes. Moreover, the intercept of Egger’s test was significantly positive *b_0_* = 0.83, *SE_b_* = 0.41, *z* = 2.03, *p* = .04. In the original article, we also assessed publication bias using a 3-parameter selection model. Assuming the presence of publication bias improved the fit of the model significantly, χ^2^(1) = 6.22, p = .012, and the fitted a 3-parameter model returned a non-significant bias-corrected mean effect of –0.14, 95% CI [–0.46, 0.16] (before –0.10, 95% CI [–0.31, 0.10]).

**Figure 2:**
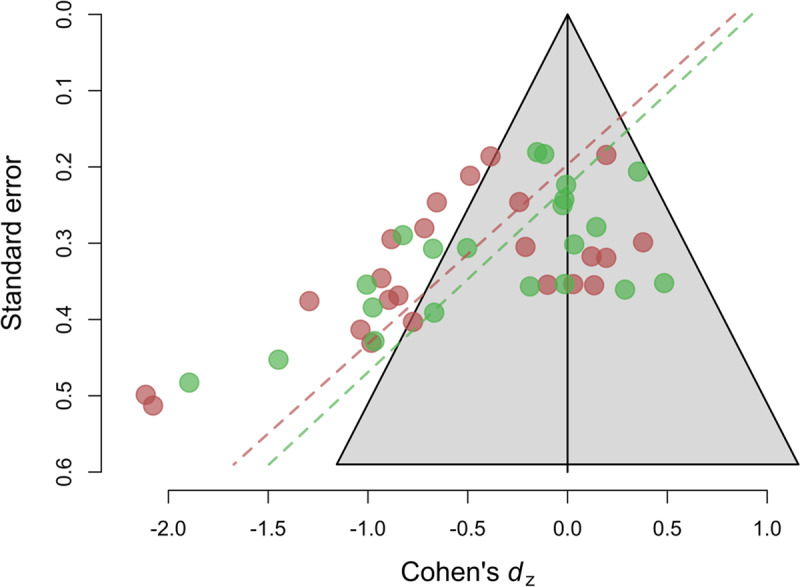
Funnel plot of Cohen’s dz effect size versus study standard error.

Likewise, for RPE, Egger’s regression test revealed significant evidence of funnel plot asymmetry, *b*_1_ = –4.1, *SE_b_* = 1.15, *z* = –3.56, p = .001, suggesting, again, that the meta-analytic average is likely to be biased by the selective publication of significant results. The intercept of Egger’s regression test was also significantly positive, *b*_0_ = 0.92, *SE_b_* = 0.35, *z* = 2.61, *p* < .014. The bias-corrected average provided by the 3-parameter selection model was small, *d*_z_ = –0.15, and non-significantly different from zero, 95% CI [–0.59, 0.27], (before *d*_z_ = –0.13 95% CI [–0.61, 0.34]) although in this case, the model assuming publication bias did not perform significantly better than the standard random-effects model, χ^2^(1) = 0.64, p = .422.
