# Supplementary material for: Mental Fatigue Might Be Not So Bad for Exercise Performance After All: A Systematic Review and Bias-Sensitive Meta-Analysis
Source: J Cogn. 2020 Oct 9;3(1):38. doi: 10.5334/joc.126 (PMC7546119; doi:10.5334/joc.126)
Supplement: Table 1. — Studies examining the effects of mental fatigue on exercise performance. [file joc-3-1-126-s1.pdf]

Table 1. Studies examining the effects of mental fatigue on exercise performance. Studies not included in quantitative meta-analysis are marked with *. TTE: Time to exhaustion test; TT: Time-trial; MVC: Maximal voluntary contraction; LSPT: Loughborough soccer passing test.

Reference	Participants	N	Experimental task	Control task	Type of exercise	Test	Measure	↓Performance	↑RPE	Mental fatigue
**Marcora et al. (2009)**	**Recreational**	**18**	**90min-AX-CPT**	**90min-documentary**	**Externally-paced**	**Cycling TTE 80%**	**Time**	**Yes**	**Yes**	**Yes**
**Brownsberger et al. (2013) ***	**Recreational**	**12**	**90min-AX-CPT**	**90min-documentary**	**Self-paced**	**Cycling TT-10min RPE 15**	**Power output**	**Yes**	**-**	**Yes**
**Pageaux et al. (2013)**	**Recreational**	**8**	**90min-AX-CPT**	**90min-documentary**	**Strength**	**TTE MVC**	**Time**	**Yes**	**Yes**	**Yes**
**MacMahon et al. (2014)**	**Well-trained**	**16**	**90min-AX-CPT**	**90min-documentary**	**Self-paced**	**Running TT-3km**	**Time**	**Yes**	**No**	**No**
**Pageaux et al. (2014)**	**Recreational**	**20**	**30min-Stroop**	**30min-Stroop-control**	**Self-paced**	**Running TT-5km**	**Time**	**Yes**	**Yes**	**No**
**Duncan et al. (2015)**	**Well-trained**	**8**	**40min-Sustained Attention**	**40min-documentary**	**Maximal Effort**	**Cycling 30seg Wingate**	**Power output**	**No**	**No**	**Na**
**Martin et al. (2015)**	**Well-trained**	**16**	**90min-AX-CPT**	**90min-documentary**	**Maximal Effort**	**Cycling 3min all-out**	**Power output**	**No**	**No**	**No**

**Smith et al. (2015)**	**Well-trained**	**10**	**90min-AX-CPT**	**90min-documentary**	**Self-paced**	**Running TT-45min**	**Distance**	**Yes**	**Yes**	**Yes**
**Azevedo et al. (2016)**	**Recreational**	**12**	**90min-AX-CPT**	**90min-documentary**	**Externally-paced**	**Cycling TTE 80%**	**Time**	**No**	**No**	**No**
**Badin et al. (2016)**	**Elite**	**12**	**30min-Stroop**	**30min-documentary**	**Soccer Specific**	**Small side game**	**Distance**	**No**	**No**	**No**
**Head et al. (2016)**	**Recreational**	**17**	**52min-Go/NoGo**	**52min-documentary**	**Strength**	**20min Body resistance exercise**	**Repetitions**	**Yes**	**-**	**Yes**
**Martin et al. (2016)**	**Elite**	**11**	**30min-Stroop**	**10 min seated**	**Self-paced**	**Cycling TT-20min**	**Power output**	**No**	**No**	**No**
**Martin et al. (2016)**	**Recreational**	**9**	**30min-Stroop**	**10 min seated**	**Self-paced**	**Cycling TT-20min**	**Power output**	**Yes**	**No**	**Yes**
**Smith et al. Exp1 (2016)**	**Recreational**	**12**	**30min-Stroop**	**30min-seated**	**Externally-paced**	**Running (Yo-Yo test)**	**Distance**	**Yes**	**Yes**	**Yes**
**Smith et al. Exp2 (2016)**	**Well-trained**	**14**	**30min-Stroop**	**30min-seated**	**Soccer Specific**	**LSPT**	**Time**	**No**	**-**	**Yes**

**Otani et al (2017)**	**Recreational**	**20**	**90min Stroop/ Sternberg/Rapid visual information**	**90min-documentary**	**Externally-paced**	**Cycling TTE 80%**	**Time**	**Yes**	**No**	**Yes**
**Van Cutsem et al. (2017) ***	**Well-trained**	**10**	**45min-Stroop**	**45min Documentary**	**Externally-paced**	**Cycling TTE-15min 80%**	**Time**	**No**	**No**	**Yes**
**Veness et al. (2017)**	**Elite**	**10**	**30min-Stroop**	**30min reading**	**Externally-paced**	**Running (Yo-Yo test)**	**Distance**	**Yes**	**Yes**	
**Vrijkotte et al. (2018) ***	**Well-trained**	**9**	**90min-Stroop**	**90min-seated**	**Externally-paced**	**Cycling Incremental test**	**Power output**	**No**	**No**	**Yes**
**Brown et al. (2019)**	**Recreational**	**25**	**50min-AX-CPT**	**50min documentary**	**Self-paced**	**Cycling TT-30min**	**Total work (Kj)**	**Yes**	**No**	**Yes**
**Clark et al. (2019)**	**Well-trained**	**10**	**30min-Stroop**	**30 min documentary**	**Self-paced**	**Cycling TT-6min**	**Total work (Kj)**	**No**	**-**	**Na**
**Clark et al. (2019)**	**Recreational**	**10**	**30min-Stroop/n-back**	**30 min documentary**	**Self-paced**	**Cycling TT-6min**	**Total work (Kj)**	**No**	**.**	**Na**
**Filipas et al. (2018)**	**Well-trained**	**8**	**60min-Stroop**	**60min-painting**	**Self-paced**	**Rowing TT-1500m**	**Time**	**No**	**No**	**Yes**

Penna et al. (2018)-a	Well-trained	8	30min-Stroop	30min-documentary	Self-paced	Swimming TT-1500m	Time	Yes	No	Yes
Penna et al. (2018)-b *	Well-trained	12	30min-Stroop	30min-documentary	Externally-paced	Running Yo-Yo test	Distance	Yes	-	Yes
Pires et al. (2018)	Recreational	12	30min Rapid Visual Information Processing (RVP) Test	30min-seated	Self-paced	Cycling TT-20km	Power output	Yes	Yes	Yes
Silva-Cavalcante et al. (2018)	Well-trained	8	90min-AX-CPT	90min-documentary	Self-paced	Cycling TT_4km	Time	No	No	Yes
Slimani et al. (2018) *	Well-trained	10	30min-Stroop	30min reading	Externally-paced	Running (shuttle test)	Distance	Yes	Yes	Yes
Staiano et al. (2018)]	Elite	13	60min-Stroop	60min-documentary	Self-paced	Kayaking TT-2000m	Time	Yes	No	Yes
Salam et al. (2018) *	Well-trained	10	30min-Stroop	30min-seated	Externally-paced	Cycling TTE	Time	Na	-	Yes
MacMahon et al. (2019)	Recreational	13	30min-Stroop	30min-Stroop-control	Externally-paced	Running shuttle test	Time	Yes	No	Yes

**Holgado et al. (2020)**	**Recreational**	**30**	**90min-AX-CPT**	**90min-documentary**	**Externally-paced**	**Cycling TTE**	**Time**	**No**	**No**	**Yes**
**Lopes et al. (2020)**	**Elite**	**31**	**45min Stroop**	**45min-documentary**	**Externally-paced**	**Running TTE**	**Time**	**Yes**	**No**	**Yes**
